# Neuropathology of yellow fever autopsy cases

**DOI:** 10.1186/s40794-022-00187-1

**Published:** 2023-01-15

**Authors:** Fernando Pereira Frassetto, Sergio Rosemberg

**Affiliations:** 1 Department of Pathology, Faculdade de Medicina da Universidade de Sao Paulo, São Paulo, SP Brazil; 2 Present Address: Department of Radiation Oncology, Ohio State University, OH Columbus, United States of America

**Keywords:** Yellow Fever, Autopsy, Brain, Neuropathology, Sepsis

## Abstract

**Background:**

Yellow fever is a viral hemorrhagic fever caused by yellow fever virus, a mosquito-borne flavivirus. Despite an effective vaccine, major outbreaks continue to occur around the world. Even though it is not a proven neurotropic virus, neurological symptoms in more severe clinical forms are frequent. The understanding of this apparent paradox is still rarely addressed in literature.

**Methods:**

The brains of thirty-eight patients with yellow fever confirmed by RT-PCR, who underwent autopsy, were analyzed morphologically to identify and characterize neuropathological changes. The data were compared with brains collected from individuals without the disease, as a control group. Both cases and controls were subdivided according to the presence or absence of co-concurrent septic shock, to exclude changes of the sepsis associated encephalopathy. To verify possible morphological differences between the yellow fever cases groups, between the control groups, and between the cases and the controls, we applied the statistical tests Fisher's exact test and chi-square, with *p* values < 0.05 considered statistically significant.

**Results:**

All cases and controls presented, at least focally, neuropathological changes, which included edema, meningeal and parenchymal inflammatory infiltrate and hemorrhages, and perivascular inflammatory infiltrate. We did not find an unequivocal aspect of encephalitis. The only parameter that, after statistical analysis, can be attributed to yellow fever was the perivascular inflammatory infiltrate.

**Conclusions:**

The neuropathological findings are sufficient to justify the multiple clinical neurologic disturbances detected in the YF cases. Since most of the parameters evaluated did not show statistically significant difference between cases and controls, an explanation for most of the neuropathological findings may be the vascular changes, consequent to shock induced endotheliopathy, associated with stimulation of the immune system inherent to systemic infectious processes. The statistical difference obtained in yellow fever cases regarding perivascular infiltrate can be can be explained by the immune activation inherent to the condition.

**Supplementary Information:**

The online version contains supplementary material available at 10.1186/s40794-022-00187-1.

## Background

Yellow fever (YF) is a viral hemorrhagic fever caused by yellow fever virus (YFV), a mosquito-borne flavivirus [1]. The largest series of YF cases descriptions in the literature come from epidemics in Africa [2–5]. Is considered a reemerging disease and, despite an effective vaccine [6], in recent years major outbreaks have been documented around the world, as occurred in Brazil between 2016 and 2018, with more than 2000 cases, including more than 500 deaths [7, 8]. The Clinics Hospital of the University of Sao Paulo School of Medicine attended individuals affected by the most severe forms of YF. Being a disease spread across the globe, often neglected, which can present severe clinical forms and even death, descriptions of pathological changes can help to determine in more detail its pathophysiology. With this aim, as some of these cases evolved with liver failure and death, autopsies were required and performed. In this way, they can contribute to advances in the clinical management of severely ill patients. [9–12].

Neurological signs and symptoms as part of the clinical picture of YF have been described for almost a century [13]. Metabolic alterations, such as hypoglycemia, hypotension, acidosis, hyperammonemia, and in the extracellular concentrations of sodium and potassium, were cited as probable causes of encephalopathy. On the other hand, direct action of YFV was considered not significant since the virus very rarely infiltrates the central nervous system [14].

Despite the common neurologic signals documented in YF cases, histopathological descriptions of the CNS in human cases are rare in the literature. Almost a century ago, Jakob showed meningeal and perivascular lymphomononuclear inflammatory infiltrate, microglial reactivity, neuronal loss, and gliosis in 14 YF cases [15]. Ten years later, Stevenson described 34 cases, reporting perivascular hemorrhage, perivascular lymphocytic infiltrate, and reactional changes in microglia and astrocytes [16].

Descriptions such as those however, are exceptions. Most studies on the neuropathology of YF are restricted to rare previously vaccinated cases (whose findings may not correspond exactly to the alterations present in the infection by wild YFV) [17], including recent descriptions of fatal meningoencephalitis in children [18, 19], and to animal models [20–23].

Therefore, our goal is to describe the neuropathology of autopsy YF cases and compare the findings to those of selected controls with fatal outcome secondary to other disturbances (septic shock, cardiovascular diseases), in an observational, case–control study.

## Methods

### Cohort description

Between December 2017 and April 2018, 38 YF cases, defined by at least one of the Brazilian Ministry of Health definition criteria—isolation of the YF virus, detection of the viral genome, detection of IgM class antibodies by the MAC-ELISA technique in unvaccinated individuals, or with a fourfold or greater increase in antibody titers by the hemagglutination inhibition (HI) technique, in paired samples [24], underwent autopsy, at University of Sao Paulo, after informed consent from first-degree relatives or legal representatives. The cases obtained were those whose autopsy had been requested by the attending physicians during the YF epidemic. The autopsies, including brain removal, followed established protocols [25, 26] and were performed by a pathologist (FPF). Of these cases, 34 were men aged between 16 and 71 years, and four were women aged between 31 and 74 years old (mean age = 48.35 years). Thirty-one presented acute neurologic disturbances reported in medical records—drowsiness, confusion, lethargy, agitation, reduced level of consciousness and/or coma. In three of them the descriptive term “encephalopathy” was used, unspecified, and in another three the designation “hepatic encephalopathy” (HE) was used. Convulsive episodes were present in 10 of them. In seven cases, detailed neurological changes were not described in the medical records.

It was possible to perform at least one electroencephalography in 21 YF cases. In 13 of them, the patient was in a coma (spontaneous or induced by sedation). In one case who had no clinically reported seizures, epileptiform paroxysms were identified. In another case alterations suggestive of toxic-metabolic and infectious encephalopathies (occasional paroxysms of acute waves, of generalized projection, outlining a triphasic aspect) were described in a report, although he did not have a detailed description of neurological signs and symptoms during evolution.

In the evaluation by in vivo computed tomography exams, performed in nine cases, three presented diffuse brain edema, one of them after a seizure episode, another presenting, in addition to edema, signs of transforaminal herniation.

All cases had as final cause of death fulminant hepatitis. Of these, 12 had an established, co-concurrent septic shock related to secondary infections, according to definition criteria [27], with at least one infectious agent isolated in cultures – *Staphylococcus* spp*, Klebsiella pneumoniae*, *Acinetobacter* spp, *Pseudomonas* spp*, Stenotrophonomas maltophilia*, *Escherichia coli*, *Micrococcus* spp, *Listeria monocytogenes*, *Candida krusei*.

For means of comparison, 21 individuals from the autopsy routine, aged between 18 and 74 years (mean age = 52 years), without YFV infection were selected by chance as controls, 10 of them with infection and septic shock, information obtained from clinical data and/or from autopsy material. This selection was based on the availability of cases without YF in the period, whose autopsy was performed after informed consent from family members or legal representatives. The inclusion and exclusion criteria of cases and controls are described in details [see Additional files 1].

### Case processing and sampling

After brain removal, in 33 cases 100 mm^2^ of the right frontal pole was sectioned and stored for reverse transcription-polymerase chain reaction (qRT-PCR) assays to detect YFV-RNA. Briefly, the tissue samples were macerated and nucleic acid extraction was performed using the TRIzol® reagent (Life Technologies). Molecular detection of YF virus was performed with the use of the AgPath-ID One-Step qRT-PCR Reagents (Ambion, Austin, TX, USA) with specific primers/probes previously described [9]. qRT-PCR reactions consisted of a step of reverse transcription at 45 °C for 10 min for enzyme activation, at 95 °C for ten minutes, and 40 cycles at 95 °C for 15 s and 60 °C for 45 s for hybridization and extension with the use of ABI7500 equipment (Thermo Fisher Scientific, Waltham, MA, USA).

Then, the brains were stored in buffered formaldehyde solution for twenty-one days, after which they were sectioned in 10 mm thickness slices. One sample on each side (symmetrical) were obtained from the frontal, parietal, occipital, temporal (mesial and lateral), basal nuclei, thalamus, centrum semiovale and cerebellar hemispheres, and one sample of each of vermis, midbrain, pons, and medulla oblongata. The samples, 30 mm large and 5 mm thick, were transferred to a histological cassette for further processing. Briefly, the samples were dehydrated in alcohol, cleaned with xylene and paraffin-embedded. The paraffin was then removed, the blocks were cooled and 5 µm-thick slices were obtained in the microtome. The slices were transferred to slides in a warm bath, dried and hematoxylin and eosin staining were performed.

### Histopathological analysis

Cases and controls were evaluated for perivascular and parenchymal edema, eosinophilic neuronal alterations, chronic small vessels changes, parenchymal hemorrhage and inflammatory infiltrate, microglial nodules, and periventricular inflammatory infiltrate, as absent (grade 0) or present (grade 1). Perivascular hemorrhage, hemosiderin deposition, and inflammatory infiltrate, meningeal hemorrhage and inflammatory infiltrate were semi-quantitatively evaluated as absent (grade 0) or present (grades 1 to 3, detailed in Table 1), an approach similar to that undertaken by Sharshar and colleagues to assess the neuropathology of septic shock [28].

**Table 1:** Adopted criteria for selected histopathological parameters grading

Parameter	Grade 1	Grade 2	Grade 3
Perivascular hemorrhage	One vessel, non-circumferential, 200 × magnification	One vessel, circumferential, 200 × magnification	Multiple vessels, 200 magnification
Perivascular hemosiderin	One vessel, non-circumferential, 200 × magnification	One vessel, circumferential, 200 × magnification	Multiple vessels, 200 magnification
Perivascular Inflammatory Infiltrate	One vessel, non-circumferential, 200 × magnification	One vessel, circumferential, 200 × magnification	Multiple vessels, 200 magnification
Meningeal Hemorrhage	One focus, 200 × magnification	Multiple foci, 200 × magnification	Focal/multifocal, 100 × magnification
Meningeal Inflammatory Infiltrate	One focus, 200 × magnification	Multiple foci, 200 × magnification	Focal/multifocal, 100 × magnification

### Statistical analysis

For the statistical analysis four groups were established: YF cases without septic shock (*n* = 26), YF cases with septic shock (*n* = 12), controls without septic shock (*n* = 11), and controls with septic shock (*n* = 10). For each YF case and each control, 13 graduation values representing the 22 mapped areas (only the highest value being considered when bilateral) were obtained. For comparison purposes, YF cases and the control groups were paired separately, and then all YF cases were compared with all controls. *p* values < 0.05 were considered statistically significant.

To verify the existence of difference (or relationship) between the YF cases groups, between the control groups, and between the YF cases and the controls, we applied the statistical tests Fisher's exact test and chi-square.

## Results

Thirty-two cases tested positive for viral genetic material by qRT-PCR, and one case tested negative. In this case, the YFV infection was confirmed by qRT-PCR and IgM in a blood sample. The five cases without tissue confirmation were positive for YFV by qRT-PCR in blood.

**Fig. 1:**
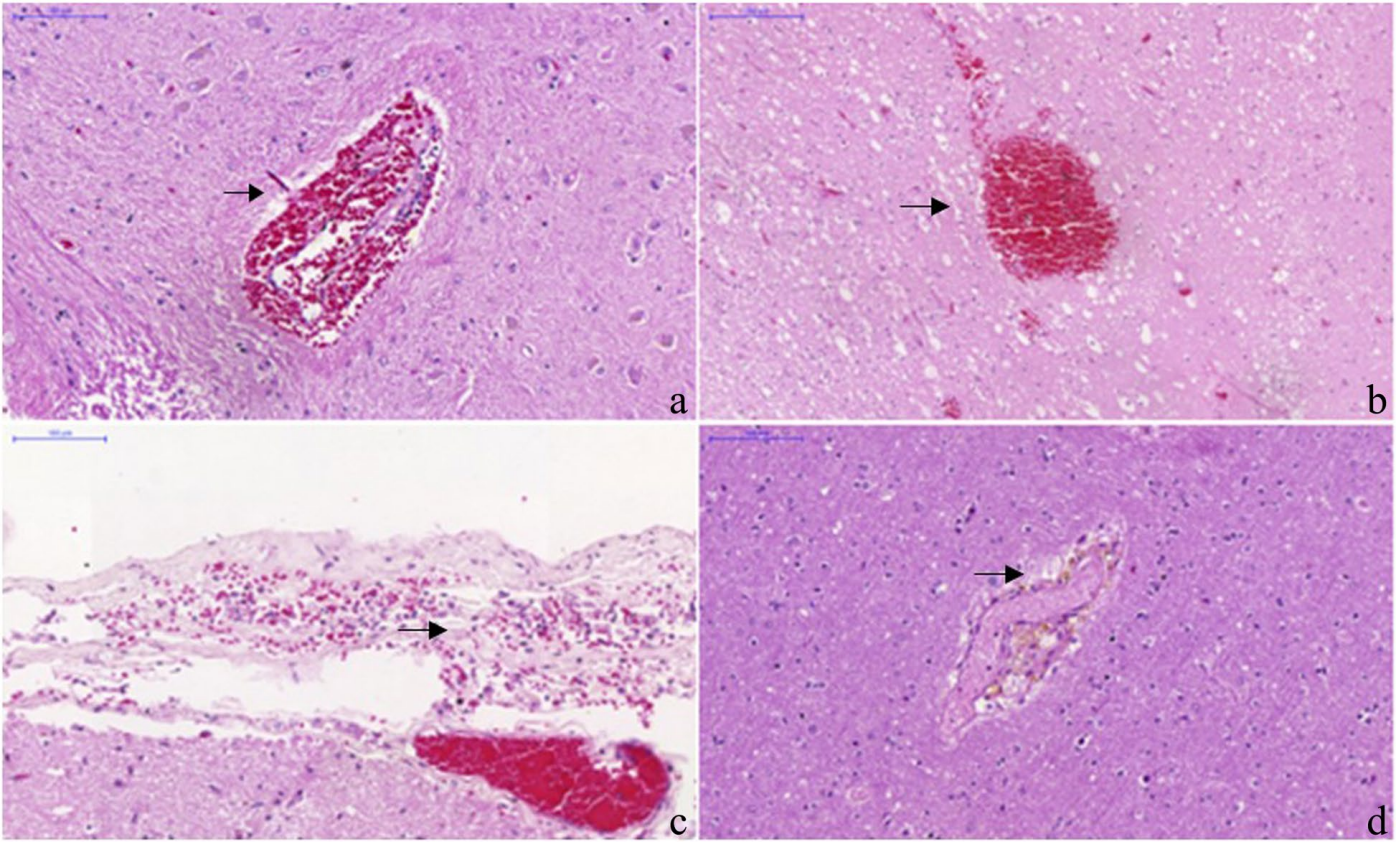
Yellow fever cases findings (hematoxylin and eosin stain). **a** Perivascular hemorrhage (black arrow), grade 2. (200 × magnification) **b** A focus of parenchymal hemorrhage (black arrow). (100x). **c** Meningeal hemorrhage (black arrows), grade 2. (200x). **d** Perivascular hemosiderin deposition black (arrow), grade 2. (100x)

**Fig. 2:**
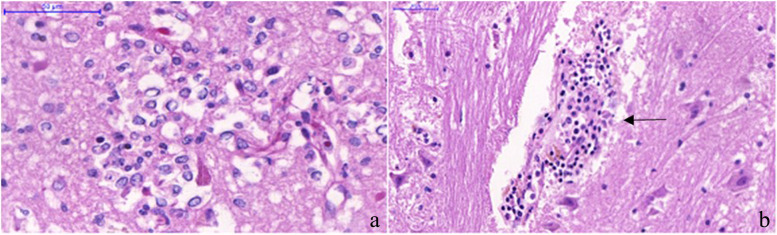
Yellow fever cases findings ((hematoxylin and eosin stain). **a** Parenchymal microglial nodule. (480 × magnification). **b** A cuff of perivascular inflammatory infiltrate (black arrow), grade 2. (400x)

The results of the evaluation of the histopathological parameters are shown in Table 2. Some examples of morphological findings can be seen in Figs. 1 and 2.

**Table 2:** Number of cases and controls with morphological alterations

Parameter	Cases (*n* = 38)	Controls (*n* = 21)
Parenchymal edema	38	21
Perivascular edema	38	21
Eosinophilic neuronal alterations	38	21
Small vessel changes	38	21
Parenchymal hemorrhage	18	13
Parenchymal inflammatory infiltrate	5	6
Perivascular hemorrhage	38	21
Perivascular inflammatory infiltrate	38	21
Perivascular hemosiderin deposition	38	21
Periventricular inflammatory infiltrate	2	0
Microglial nodules	5	1
Meningeal hemorrhage	38	21
Meningeal inflammatory infiltrate	38	21

When comparing the YF cases groups, there was only statistical difference related to perivascular hemorrhage (higher number of samples with grades 2 and 3 perivascular hemorrhage, *p* = 0.006) and microglial nodules (higher number of cases with nodules, *p* = 0.031) were observed the cases with concomitant septic shock. When comparing the control groups, there was no statistically significant difference in any histopathological parameter.

When comparing the YF cases and the controls, significant difference was obtained for four parameters. Samples with grades 0 or 1 perivascular hemorrhage were more prevalent in the YF cases than in controls, the inverse occurring in grades 2 and 3 (*p* < 0.0001). A higher number of samples with parenchymal hemorrhage was obtained in the controls (*p* < 0.0001). A higher number of samples with perivascular inflammatory infiltrate was obtained in the YF cases (*p* < 0.0001). A higher number of grade 1 meningeal hemorrhage samples was obtained in the cases compared to controls, the opposite occurring for grades 2 and 3 (*p* < 0.0001).

## Discussion

Acute neurological disturbances are commonly related in YF patients, but its pathophysiology remains obscure. To clarify this issue, we performed a neuropathological study of 38 YF patients who progressed to death and compared with 21 controls with death not related to YF. Both groups were subdivided according the presence or absence of septic shock, for this condition by itself may be the cause of neurological disturbances. The so-called sepsis associated encephalopathy has been shown to be associated with diffuse cerebral damage and specific autonomic neuronal apoptosis which may be due to circulating factors particularly inducible nitric oxide synthetase (iNOS) [28]. It is known that even milder infections, without defined shock, can cause neurological changes, and that shock from other etiologies, by itself, is a factor associated with marked vascular disturbances, related to a severe endotheliopathy [29, 30].

The so-called shock induced endotheliopathy (SHINE) is characterized by severe hemostatic aberrations and coagulopathy, which are associated with excess mortality, being the shock-induced sympatho-adrenal hyperactivation a critical driver of endothelial cell and glycocalyx damage (endotheliopathy) in acute critical illness, with the overall aim of ensuring organ perfusion through an injured microvasculature [31].

Statistically, when comparing the YF cases groups with and without septic shock, significant difference was obtained for the perivascular hemorrhage and microglial nodules parameters. Regarding the intensity of perivascular hemorrhages, it can be said that the presence of YF and septic shock acted in a synergistic way, which can be explained by the marked coagulopathy, usually as a complex condition that can be either hypocoagulability or a procoagulant state, observed in the context of various infectious diseases, and especially evident in viral hemorrhagic fevers [32–35]. Regarding microglial nodules, the difference can be attributed to the more intense activation of the immune system in the presence of concomitant (viral and bacterial) infections.

When comparing the YF cases and the controls, significant difference was obtained in relation to parenchymal and meningeal hemorrhages, as well as to the perivascular inflammatory infiltrate. It was noted that, in the controls, parenchymal hemorrhage was present in more samples, and the hemorrhages in other compartments were more intense. It is known that, in conditions of hemodynamic instability, the brain must continue to receive adequate blood flow, for example by vasodilation [36]. In view of our observation that the hemorrhagic phenomena were more intense in the controls, which often had a fulminant condition (generally less than 24 h), that resulted in death, we can attribute such more intense hemorrhagic phenomena to the transient and abrupt increase in perfusion, in an altered vascular environment (e.g., small vessels alterations), resulting in less ability to manage sudden pressure changes. On the other hand, a higher percentage of samples with perivascular inflammatory infiltrate in the YF cases can be explained by the disease itself, from the immune activation inherent to the condition.

It therefore seems evident that the changes present in the CNS of YF cases are mainly related to a critical patient condition, with multiple organ failure, especially the liver, associated mainly with severe vascular disturbances.

## Conclusions and future directions

The multiple neuropathological findings, many of which are in line with previous works, including non-significant encephalitis [15, 16], are sufficient to justify the neurologic disturbances detected in the YF cases. According to the fact that shock (septic or other etiology) was invariably present in our series, we can justify the findings by the association of dysfunction of the vascular system and stimulation of the immune system by YFV (and other infectious agents, when present), in accordance with descriptions of endothelial changes in shock [29–31]. The absence of significant encephalitis reinforces these vascular mechanisms as the main responsible for the observed changes in the YF cases.

In view of the findings described, it is important for the care team that, in cases of severe yellow fever, strict observance of circulatory and coagulation parameters is carried out, to maintain good brain perfusion, at the same time avoiding hemorrhagic phenomena, in an attempt to avoid neurological damage and even death.

It is also important that further studies addressing the neuropathological changes in fatal cases of YF are carried out, emphasizing the relevance of the autopsy as an instrument of knowledge and provider of information that may be relevant for multidisciplinary care in future epidemics. As an often-neglected disease with known epidemiological patterns, such studies will be of great relevance to the management of severe and potentially fatal cases.

## Supplementary Information

**Additional file 1.** Inclusion and exclusion criteria of cases and controls.

## Data Availability

The dataset supporting this article is available upon request; please contact the corresponding author.

## Abbreviations

YF: Yellow Fever
YFV: Yellow Fever Virus
RT-PCR: Reverse Transcription Polymerase Chain Reaction
RNA: Ribonucleic Acid
H&E: Hematoxylin and Eosin
