# Supplementary material for: Neuropathology of yellow fever autopsy cases
Source: Trop Dis Travel Med Vaccines. 2023 Jan 15;9:1. doi: 10.1186/s40794-022-00187-1 (PMC9883951; doi:10.1186/s40794-022-00187-1)
Supplement: Supplementary file 1 — Additional file 1. Inclusion and exclusion criteria of cases and controls. [file 40794_2022_187_MOESM1_ESM.docx]

**Additional files 1: Inclusion and Exclusion criteria.**

**1. Yellow fever cases**

Inclusion criteria:

. Confirmation of YFV infection, in life and/or in autopsy material, according to the definition of confirmed human case of the Brazilian Ministry of Health - Any suspected case that presents at least one of the following conditions: Isolation of the YF virus; Detection of the viral genome; Detection of IgM class antibodies by the MAC-ELISA technique in unvaccinated individuals or with a fourfold or greater increase in antibody titers by the hemaglutination inhibition (HI) technique, in paired samples.

. Death of ill-defined cause, that is, whose autopsy was considered necessary to clarify the cause of death.

. Absence of known primary neurological diseases, or that have been diagnosed during medical care.

Exclusion criteria:

. Suspected cases according to the Brazilian Ministry of Health definition but whose YFV infection has not been confirmed.

. Suspected or confirmed history of death directly related to exogenous/traumatic agents. . Individuals whose death occurred more than 24 hours before the autopsy was performed.

**2. Controls**:

Inclusion criteria:

. Death of ill-defined cause, that is, whose autopsy was considered necessary to clarify the cause of death.

. Absence of known primary neurological diseases, or that have been diagnosed during medical care.

. Age over 18 years (minimum age of cases) and under 74 years (maximum age of cases).

Exclusion criteria:

. Suspected or confirmed YF, or vaccination for it.

. Suspected or confirmed history of death directly related to exogenous/traumatic agents.

. Individuals whose death occurred more than 24 hours before the autopsy was performed.
